# *catena*-Poly[potassium-di-μ-aqua-μ-4-(5-tetra­zolio)pyridine]

**DOI:** 10.1107/S1600536809018649

**Published:** 2009-05-23

**Authors:** Li-Jing Cui

**Affiliations:** a Ordered Matter Science Research Center, College of Chemistry and Chemical Engineering, Southeast University, Nanjing 210096, People’s Republic of China

## Abstract

The title compound, [K(C_6_H_4_N_5_)(H_2_O)_2_]_*n*_, was synthesized by hydro­thermal reaction of KOH with 4-(5-tetra­zolio)pyridine. The K atom has a distorted octa­hedral coordination environment and is coordinated by two axial N atoms from the organic ligand and by four water mol­ecules in the equatorial plane. The mol­ecules as a whole are located on crystallographic mirror planes; the K atom is also located on an inversion center. Both the water mol­ecules and the organic ligands act as bridges to link symmetrically the adjacent K atoms into polymeric chains parallel to the *c* axis. O—H⋯N hydrogen bonds involving the water O atoms and aromatic π–π inter­actions [centroid–centroid distance 3.80 (2) Å] between the pyridine and tetra­zole rings build up an infinite three-dimensional network.

## Related literature

For applications of tetra­zole derivatives in coordination chemistry, see: Xiong *et al.* (2002 ▶); Wang *et al.* (2005 ▶). For the crystal structure of a related compound, see: Dai & Fu (2008 ▶).
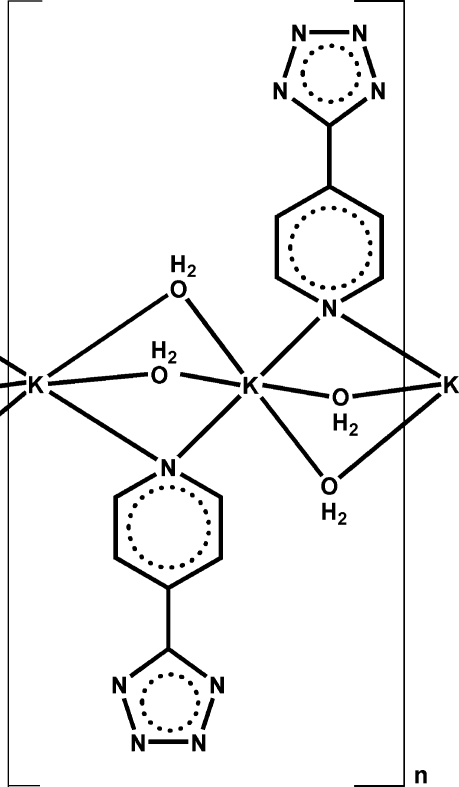


## Experimental

### 

#### Crystal data

[K(C_6_H_4_N_5_)(H_2_O)_2_]
*M* *_r_* = 221.27
Monoclinic, 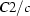

*a* = 12.361 (3) Å
*b* = 12.281 (3) Å
*c* = 7.3431 (15) Å
β = 117.25 (3)°
*V* = 991.1 (3) Å^3^
*Z* = 4
Mo *K*α radiation
μ = 0.52 mm^−1^
*T* = 298 K
0.25 × 0.15 × 0.10 mm

#### Data collection

Rigaku Mercury2 diffractometer
Absorption correction: multi-scan (*CrystalClear*; Rigaku, 2005 ▶) *T*_min_ = 0.913, *T*_max_ = 1.000 (expected range = 0.867–0.949)
5056 measured reflections
1134 independent reflections
928 reflections with *I* > 2σ(*I*)
*R*_int_ = 0.027

#### Refinement

*R*[*F* ^2^ > 2σ(*F* ^2^)] = 0.037
*wR*(*F* ^2^) = 0.096
*S* = 1.07
1134 reflections
67 parameters
2 restraints
H-atom parameters constrained
Δρ_max_ = 0.32 e Å^−3^
Δρ_min_ = −0.19 e Å^−3^

### 

Data collection: *CrystalClear* (Rigaku, 2005 ▶); cell refinement: *CrystalClear*; data reduction: *CrystalClear*; program(s) used to solve structure: *SHELXS97* (Sheldrick, 2008 ▶); program(s) used to refine structure: *SHELXL97* (Sheldrick, 2008 ▶); molecular graphics: *SHELXTL* (Sheldrick, 2008 ▶); software used to prepare material for publication: *SHELXTL*.

## Supplementary Material

Crystal structure: contains datablocks I, global. DOI: 10.1107/S1600536809018649/zl2195sup1.cif

Structure factors: contains datablocks I. DOI: 10.1107/S1600536809018649/zl2195Isup2.hkl

Additional supplementary materials:  crystallographic information; 3D view; checkCIF report

## Figures and Tables

**Table 1:** Hydrogen-bond geometry (Å, °)

*D*—H⋯*A*	*D*—H	H⋯*A*	*D*⋯*A*	*D*—H⋯*A*
O1*W*—H1*WA*⋯N2^i^	0.84	2.01	2.852 (2)	177
O1*W*—H1*WB*⋯N3^ii^	0.88	1.97	2.831 (2)	169

Symmetry codes: (i) 
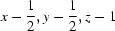
; (ii) 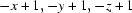.

**Fig. 1.**
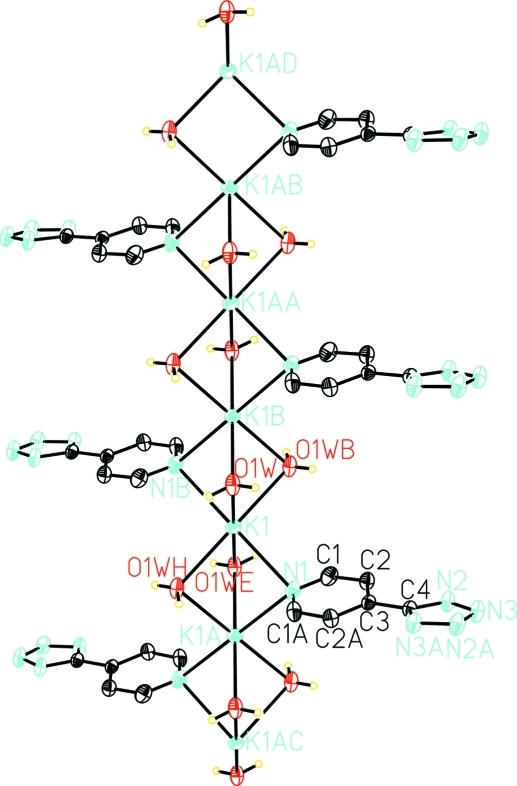
A view of the title compound with the atomic numbering scheme. Displacement ellipsoids were drawn at the 30% probability level.

**Fig. 2.**
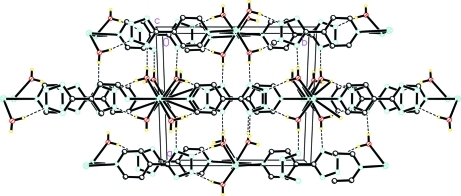
The crystal packing of the title compound viewed along the c axis showing the three dimensionnal network (dashed lines). Hydrogen atoms not involved in hydrogen bonding have been omitted for clarity.

## supplementary crystallographic information

### Comment

In the past few years, more and more people have focused on the chemistry of tetrazole derivatives because of their multiple coordination modes as ligands to metal ions and for the construction of novel metal-organic frameworks (Wang, *et al.* 2005; Xiong, *et al.* 2002). We report here the crystal structure of the title compound, tetra-aqua-bis[4-(2*H*-tetrazol-5-yl)pyridine]potassium(I).

The K atom has a distorted octahedral geometry and is coordinated by two axial pyridyl N atoms from the organic ligand and four water molecules ligands in the equatorial plane. The molecules as a whole are located on crystallographic mirror planes, the potassium ion is also located on an inversion center. Both the water molecules and the organic ligands act as bridges linking adjacent K ions into polymeric chains parallel to the *c* axis by covalent bonds (K—N, and K—O). The pyridine and tetrazole rings are nearly coplanar and are twisted from each other by a dihedral angle of only 12.99 (0.13) ° (Fig.1). The bond distances and bond angles of the tetrazole rings are in the usual ranges (Wang, *et al.* 2005; Dai & Fu 2008).

The crystal packing (Fig. 2) is stabilized by aromatic π–π interactions between the pyridine and tetrazole rings of the neighbouring ligand systems. The centroid···centroid distance is 3.80 (2)Å (symmetry code: x, y, z+1 and x, y, z). The molecular packing is further stabilized by intermolecular O—H···N hydrogen bonds involving the aqueous O atoms. The π–π and hydrogen bonding interactions build up an infinite three-dimensional network. (Fig. 2 and Table 1).

### Experimental

A mixture of 4-(2*H*-tetrazol-5-yl)pyridine (0.4 mmol) and KOH (0.4 mmol), ethanol (1 ml) and a few drops of water sealed in a glass tube was maintained at 373 K. Colorless needle crystals suitable for X-ray analysis were obtained after 3 days.

### Refinement

All H atoms attached to C atoms were fixed geometrically and treated as riding with C–H = 0.93 Å (aromatic) with *U*_iso_(H) = 1.2Ueq(C). The H atoms of water molecules were located in difference Fourier maps and the O–H distances were restrained in the subsequent refinements to 0.85 Å with *U*_iso_(H) =1.5Ueq(O). In the last stage of the refinement they were treated as riding on the O atom.

### Crystal data

**Table:**

[K(C_6_H_4_N_5_)(H_2_O)_2_]	*F*(000) = 456
*M**_r_* = 221.27	*D*_x_ = 1.483 Mg m^−^^3^
Monoclinic, *C*2/*c*	Mo *K*α radiation, λ = 0.71073 Å
Hall symbol: -C 2yc	Cell parameters from 1134 reflections
*a* = 12.361 (3) Å	θ = 3.3–27.5°
*b* = 12.281 (3) Å	µ = 0.52 mm^−^^1^
*c* = 7.3431 (15) Å	*T* = 298 K
β = 117.25 (3)°	Needle, colorless
*V* = 991.1 (3) Å^3^	0.25 × 0.15 × 0.10 mm
*Z* = 4	

### Data collection

**Table:**

Rigaku Mercury2 (2× 2 bin mode) diffractometer	1134 independent reflections
Radiation source: fine-focus sealed tube	928 reflections with *I* > 2σ(*I*)
graphite	*R*_int_ = 0.027
Detector resolution: 13.6612 pixels mm^-1^	θ_max_ = 27.5°, θ_min_ = 3.3°
ω scans	*h* = −16→16
Absorption correction: multi-scan (*CrystalClear*; Rigaku, 2005)	*k* = −15→15
*T*_min_ = 0.913, *T*_max_ = 1.000	*l* = −9→9
5056 measured reflections	

### Refinement

**Table:**

Refinement on *F*^2^	Primary atom site location: structure-invariant direct methods
Least-squares matrix: full	Secondary atom site location: difference Fourier map
*R*[*F*^2^ > 2σ(*F*^2^)] = 0.037	Hydrogen site location: inferred from neighbouring sites
*wR*(*F*^2^) = 0.096	H-atom parameters constrained
*S* = 1.07	*w* = 1/[σ^2^(*F*_o_^2^) + (0.039*P*)^2^ + 0.7641*P*] where *P* = (*F*_o_^2^ + 2*F*_c_^2^)/3
1134 reflections	(Δ/σ)_max_ < 0.001
67 parameters	Δρ_max_ = 0.32 e Å^−^^3^
2 restraints	Δρ_min_ = −0.19 e Å^−^^3^

### Special details

**Table:**

Geometry. All e.s.d.'s (except the e.s.d. in the dihedral angle between two l.s. planes) are estimated using the full covariance matrix. The cell e.s.d.'s are taken into account individually in the estimation of e.s.d.'s in distances, angles and torsion angles; correlations between e.s.d.'s in cell parameters are only used when they are defined by crystal symmetry. An approximate (isotropic) treatment of cell e.s.d.'s is used for estimating e.s.d.'s involving l.s. planes.
Refinement. Refinement of *F*^2^ against ALL reflections. The weighted *R*-factor *wR* and goodness of fit *S* are based on *F*^2^, conventional *R*-factors *R* are based on *F*, with *F* set to zero for negative *F*^2^. The threshold expression of *F*^2^ > σ(*F*^2^) is used only for calculating *R*-factors(gt) *etc*. and is not relevant to the choice of reflections for refinement. *R*-factors based on *F*^2^ are statistically about twice as large as those based on *F*, and *R*- factors based on ALL data will be even larger.

### Fractional atomic coordinates and isotropic or equivalent isotropic displacement parameters (Å^2^)

**Table:**

	*x*	*y*	*z*	*U*_iso_*/*U*_eq_	
K1	0.5000	0.0000	0.5000	0.0467 (2)	
C4	0.5000	0.5325 (2)	0.7500	0.0365 (6)	
C3	0.5000	0.4124 (2)	0.7500	0.0358 (5)	
N2	0.60000 (14)	0.59305 (13)	0.8162 (3)	0.0468 (4)	
C2	0.60283 (18)	0.35391 (16)	0.7821 (3)	0.0459 (5)	
H2	0.6742	0.3897	0.8050	0.055*	
N3	0.55965 (15)	0.69599 (13)	0.7896 (3)	0.0530 (5)	
N1	0.5000	0.18446 (19)	0.7500	0.0522 (6)	
C1	0.5980 (2)	0.24208 (17)	0.7797 (4)	0.0534 (5)	
H1	0.6678	0.2041	0.8000	0.064*	
O1W	0.34559 (12)	0.08972 (11)	0.1308 (2)	0.0516 (4)	
H1WA	0.2731	0.0885	0.0381	0.077*	
H1WB	0.3654	0.1588	0.1421	0.077*	

### Atomic displacement parameters (Å^2^)

**Table:**

	*U*^11^	*U*^22^	*U*^33^	*U*^12^	*U*^13^	*U*^23^
K1	0.0545 (4)	0.0460 (4)	0.0403 (3)	−0.0005 (3)	0.0221 (3)	−0.0025 (3)
C4	0.0345 (13)	0.0344 (12)	0.0323 (13)	0.000	0.0080 (11)	0.000
C3	0.0381 (13)	0.0334 (13)	0.0296 (12)	0.000	0.0102 (10)	0.000
N2	0.0383 (9)	0.0337 (8)	0.0522 (10)	−0.0025 (7)	0.0067 (7)	−0.0016 (7)
C2	0.0392 (10)	0.0406 (10)	0.0536 (12)	−0.0008 (8)	0.0176 (9)	−0.0029 (9)
N3	0.0503 (9)	0.0340 (8)	0.0541 (11)	−0.0042 (7)	0.0062 (8)	−0.0020 (7)
N1	0.0602 (16)	0.0343 (12)	0.0524 (15)	0.000	0.0174 (13)	0.000
C1	0.0514 (12)	0.0416 (11)	0.0596 (13)	0.0090 (9)	0.0189 (10)	−0.0026 (9)
O1W	0.0341 (7)	0.0376 (7)	0.0667 (10)	0.0014 (6)	0.0088 (7)	0.0026 (6)

### Geometric parameters (Å, °)

**Table:**

K1—O1W^i^	2.7309 (16)	C3—C2^iv^	1.383 (2)
K1—O1W^ii^	2.7309 (16)	C3—C2	1.383 (2)
K1—O1W	2.7330 (17)	N2—N3	1.340 (2)
K1—O1W^iii^	2.7330 (17)	C2—C1	1.375 (3)
K1—N1^iii^	2.9159 (18)	C2—H2	0.9300
K1—N1	2.9159 (18)	N3—N3^iv^	1.314 (3)
K1—C1^iii^	3.499 (2)	N1—C1	1.332 (3)
K1—C1	3.499 (2)	N1—C1^iv^	1.332 (3)
K1—K1^ii^	3.6716 (7)	N1—K1^iv^	2.9159 (18)
K1—K1^iv^	3.6716 (8)	C1—H1	0.9300
K1—H1WB	3.0780	O1W—K1^ii^	2.7309 (16)
C4—N2	1.329 (2)	O1W—H1WA	0.8412
C4—N2^iv^	1.329 (2)	O1W—H1WB	0.8765
C4—C3	1.475 (3)		
			
O1W^i^—K1—O1W^ii^	180.00 (3)	C1^iii^—K1—K1^iv^	115.36 (4)
O1W^i^—K1—O1W	103.20 (5)	C1—K1—K1^iv^	64.64 (4)
O1W^ii^—K1—O1W	76.80 (5)	K1^ii^—K1—K1^iv^	180.0
O1W^i^—K1—O1W^iii^	76.80 (5)	O1W^i^—K1—H1WB	111.4
O1W^ii^—K1—O1W^iii^	103.20 (5)	O1W^ii^—K1—H1WB	68.6
O1W—K1—O1W^iii^	180.0	O1W—K1—H1WB	16.0
O1W^i^—K1—N1^iii^	96.28 (4)	O1W^iii^—K1—H1WB	164.0
O1W^ii^—K1—N1^iii^	83.72 (4)	N1^iii^—K1—H1WB	96.4
O1W—K1—N1^iii^	83.68 (4)	N1—K1—H1WB	83.6
O1W^iii^—K1—N1^iii^	96.32 (4)	C1^iii^—K1—H1WB	97.5
O1W^i^—K1—N1	83.72 (4)	C1—K1—H1WB	82.5
O1W^ii^—K1—N1	96.28 (4)	K1^ii^—K1—H1WB	52.7
O1W—K1—N1	96.32 (4)	K1^iv^—K1—H1WB	127.3
O1W^iii^—K1—N1	83.68 (4)	N2—C4—N2^iv^	112.0 (2)
N1^iii^—K1—N1	180.0	N2—C4—C3	124.01 (11)
O1W^i^—K1—C1^iii^	75.74 (5)	N2^iv^—C4—C3	124.01 (11)
O1W^ii^—K1—C1^iii^	104.26 (5)	C2^iv^—C3—C2	117.4 (2)
O1W—K1—C1^iii^	82.15 (5)	C2^iv^—C3—C4	121.30 (12)
O1W^iii^—K1—C1^iii^	97.85 (5)	C2—C3—C4	121.30 (12)
N1^iii^—K1—C1^iii^	21.60 (4)	C4—N2—N3	104.64 (16)
N1—K1—C1^iii^	158.40 (4)	C1—C2—C3	119.1 (2)
O1W^i^—K1—C1	104.26 (5)	C1—C2—H2	120.5
O1W^ii^—K1—C1	75.74 (5)	C3—C2—H2	120.5
O1W—K1—C1	97.85 (5)	N3^iv^—N3—N2	109.38 (10)
O1W^iii^—K1—C1	82.15 (5)	C1—N1—C1^iv^	115.8 (2)
N1^iii^—K1—C1	158.40 (4)	C1—N1—K1	104.69 (11)
N1—K1—C1	21.60 (4)	C1^iv^—N1—K1	124.89 (11)
C1^iii^—K1—C1	180.00 (9)	C1—N1—K1^iv^	124.89 (11)
O1W^i^—K1—K1^ii^	132.19 (4)	C1^iv^—N1—K1^iv^	104.69 (11)
O1W^ii^—K1—K1^ii^	47.81 (4)	K1—N1—K1^iv^	78.04 (6)
O1W—K1—K1^ii^	47.76 (3)	N1—C1—C2	124.3 (2)
O1W^iii^—K1—K1^ii^	132.24 (3)	N1—C1—K1	53.71 (10)
N1^iii^—K1—K1^ii^	50.98 (3)	C2—C1—K1	149.08 (16)
N1—K1—K1^ii^	129.02 (3)	N1—C1—H1	117.8
C1^iii^—K1—K1^ii^	64.64 (4)	C2—C1—H1	117.8
C1—K1—K1^ii^	115.36 (4)	K1—C1—H1	73.4
O1W^i^—K1—K1^iv^	47.81 (4)	K1^ii^—O1W—K1	84.44 (4)
O1W^ii^—K1—K1^iv^	132.19 (4)	K1^ii^—O1W—H1WA	111.4
O1W—K1—K1^iv^	132.24 (3)	K1—O1W—H1WA	142.8
O1W^iii^—K1—K1^iv^	47.76 (3)	K1^ii^—O1W—H1WB	102.4
N1^iii^—K1—K1^iv^	129.02 (3)	K1—O1W—H1WB	105.0
N1—K1—K1^iv^	50.98 (3)	H1WA—O1W—H1WB	104.0

Symmetry codes: (i) *x*, −*y*, *z*+1/2; (ii) −*x*+1, *y*, −*z*+1/2; (iii) −*x*+1, −*y*, −*z*+1; (iv) −*x*+1, *y*, −*z*+3/2.

### Hydrogen-bond geometry (Å, °)

**Table:**

*D*—H···*A*	*D*—H	H···*A*	*D*···*A*	*D*—H···*A*
O1W—H1WA···N2^v^	0.84	2.01	2.852 (2)	177
O1W—H1WB···N3^vi^	0.88	1.97	2.831 (2)	169

Symmetry codes: (v) *x*−1/2, *y*−1/2, *z*−1; (vi) −*x*+1, −*y*+1, −*z*+1.
